# Affection of Motor Network Regions by Tau Pathology Across the Alzheimer's Disease Spectrum

**DOI:** 10.1523/ENEURO.0242-23.2023

**Published:** 2024-01-16

**Authors:** Gérard N. Bischof, Elena Jaeger, Kathrin Giehl, Merle C. Hönig, for the Alzheimer’s Disease Neuroimaging Initiative, Peter H. Weiss, Alexander Drzezga

**Affiliations:** ^1^Department of Nuclear Medicine, Faculty of Medicine and University Hospital of Cologne, University of Cologne, 50937 Cologne, Germany; ^2^Molecular Organization of the Brain, Institute for Neuroscience and Medicine II, Research Center Juelich, 52428 Juelich, Germany; ^3^Cognitive Neuroscience, Institute for Neuroscience and Medicine III, Research Center Juelich, 52428 Juelich,Germany; ^4^Department of Neurology, Faculty of Medicine and University Hospital of Cologne, Universityof Cologne, 50937 Cologne, Germany; ^5^German Center for Neurodegenerative Diseases Bonn/Cologne, 53127 Bonn, Germany

**Keywords:** Alzheimer's disease, mild cognitive impairment, regional tau burden, motor regions, positron emission tomography

## Abstract

Stereotypical isocortical tau protein pathology along the Braak stages has been described as an instigator of neurodegeneration in Alzheimer's disease (AD). Less is known about tau pathology in motor regions, although higher-order motor deficits such as praxis dysfunction are part of the clinical description. Here, we examined how tau pathology in cytoarchitectonically mapped regions of the primary and higher-order motor network in comparison to primary visual and sensory regions varies across the clinical spectrum of AD. We analyzed tau PET scans from the Alzheimer's Disease Neuroimaging Initiative (ADNI) cohort in patients with mild cognitive impairment (MCI; *N* = 84) and dementia of the Alzheimer's disease type (DAD; *N* = 25). Additionally, an amyloid-negative sample of healthy older individuals (HC; *N* = 26) were included. Standard uptake ratio values (SUVRs) were extracted in native space from the left and the right hemispheres. A repeated measurement analysis of variance was conducted to assess the effect of diagnostic disease category on tau pathology in the individual motor regions, controlling for age. We observed that tau pathology varies as a function of diagnostic category in predominantly higher motor regions (i.e., supplementary motor area, superior parietal lobe, angular gyrus, and dorsal premotor cortex) compared to primary visual, sensory and motor regions. Indeed, tau in higher-order motor regions was significantly associated with decline in cognitive function. Together, these results expand our knowledge on the in vivo pattern of tau pathology in AD and suggest that higher motor regions are not spared from tau aggregation in the course of disease, potentially contributing to the symptomatic appearance of the disease.

## Significance Statement

The presented data show relevant tau pathology in higher-order motor regions of patients with mild cognitive impairment (MCI) and dementia of the Alzheimer's disease type (DAD), in a set of regions often neglected in the previous literature. Tau accumulation in higher-order motor regions was associated with clinical disease severity and increased cognitive dysfunction. These findings suggest that the concerted vulnerability of motor regions to tau pathology may contribute to motor praxis dysfunction observed in Alzheimer's disease.

## Introduction

Alzheimer's disease (AD) pathology is characterized by the build-up of senile plaques composed of β-amyloid in the extracellular space and neurofibrillary tangles composed of tau proteins in the intracellular space. β-amyloid generally appears first in the neocortex, and then spreads to the allocortex and subcortical regions as the disease advances (Thal et al., 2002; Grothe et al., 2017). In contrast, tau has been described by a stereotypical spreading pattern starting in the transenthorinal regions, progressing to the temporal allocortex, followed by parietal and occipital high-order association areas and prefrontal regions, before spreading to the unimodal sensory and premotor areas (Braak and Braak, 1995; Braak et al., 2011). In vivo imaging using positron emission tomography (PET) of AD pathology has provided general correspondence for the histopathological distribution of both protein pathologies (Thal et al., 2002; Schöll et al., 2016; Schwarz et al., 2016; Bischof et al., 2017; Grothe et al., 2017; Jack et al., 2018), whereas the regional accumulation of tangle pathology has been shown to be stronger related to the clinical symptoms compared to β-amyloid plaques (Dronse et al., 2016; Ossenkoppele et al., 2016).

However, in vivo imaging offers the novel opportunity to evaluate if tau pathology is present in regions, which are often neglected in the staging scheme stated by histopathological studies. Indeed, for example, regions of the lateral occipital cortex were associated with tau accumulation in histopathological studies, in late disease only, but were found in mild and moderate stages of the disease (Schwarz et al., 2016) using in vivo PET. Likewise, regions of the motor system have gained little attention in histopathological studies. For instance, histopathological studies revealed β-amyloid accumulation in primary motor regions in Thal stage 4 and 5 that preceded subcortical burden of β-amyloid in regions of the hippocampus (Murray et al., 2015). Similarly, tau pathology has been described in sensorimotor cortex only in late isocortical disease stages (Schwarz et al., 2016). Yet, a systematic in vivo study on the distribution of tau pathology in regions of the motor network is missing, although it is feasible that tau in motor regions may contribute to the clinical symptomatology of AD.

Notably, there is a great deal of clinical interest in the evaluation of motor symptoms, as apraxia has been reported in patients with a clinical diagnosis of dementia of the Alzheimer's disease type (DAD) with up to 32% (Ozkan et al., 2013) and has been recognized as a symptom category that can differentiate variants of dementia based on the specific apraxia profile (Johnen et al., 2018). It is widely unknown, however, which aspects of the pathophysiological disease cascade contributes to motor deficits in AD. Specifically, it has yet to be determined whether significant tau pathology can be found in motor regions of DAD patients. Here, we probed these questions by first quantifying the in vivo tau deposition in motor regions in patients clinically diagnosed with mild cognitive impairment (MCI) and DAD patients (compared to healthy older participants). Secondly, we evaluated if the level of tau deposition in motor regions (compared to control regions) was associated with disease stage or general cognitive dysfunction.

## Material and Methods

### Participants

Data used for this study were derived from the Alzheimer's Disease Neuroimaging Initiative (ADNI) (http://adni.loni.usc.edu/). Screening criteria were the following: (1) information on amyloid status, (2) availability of a 18F-AV1451 scan, and (3) available MRI scan within an appropriate time window around 18F-AV1451 scan acquisition with the longest acceptable difference between MRI and 18F-AV1451 being 6 months (median time difference = 14 d with standard deviation (SD) = 38.17 d).

The patients with clinical dementia due to AD met the National Institute of Neurological and Communicative Disorders and Stroke–Alzheimer's Disease and Related Disorders Association criteria for probable AD (McKhann et al., 1984). Briefly, patients with DAD had progressive deterioration of memory and other cognitive domains, objectively evaluated in a clinical exam in the absence of another systemic, psychiatric or neurological disease that could potentially explain dementia symptoms. Activities of daily living (ADL) were impaired. Patients with MCI reported subjective memory concern either autonomously or via an informant or clinician and showed an objective cognitive impairment defined by education-adjusted scores with 1.5 SDs below the normative mean on delayed recall of Wechsler Memory Scale Story A. ADL were essentially preserved, and no signs of dementia existed (Petersen et al., 2010). Healthy controls (HC) in this sample showed no signs of depression did not fulfill the criteria for MCI or DAD and were amyloid-negative based on PET or CSF information derived from ADNI (https://adni.loni.usc.edu/methods/) closest to the tau PET scans.

### Experimental design and statistical analyses

#### Neuropsychological tests to assess global cognitive performance

All participants underwent neuropsychological testing using the Alzheimer's Disease Assessment Scale-Cognitive Subscale (ADAS-Cog-13). The cognitive subscale includes 13 self-completed or observer-based assessments evaluating the cognitive domains of memory, language, attention, and concentration. For our analysis, we calculated the global performance score on the ADAS-Cog-13. Of note, severity in cognitive decline is quantified by higher values on the ADAS-subscale culminating in higher scores in more progressed patients. Additionally, neuropsychological test scores of the Mini-Mental Examination Test (MMSE) were used to describe the status of global cognitive function of each participant in the groups.

#### Positron emission tomography (PET) acquisition

18F-AV1451 PET imaging was acquired using a static imaging protocol 75 min post-injection of ∼370 MBq (10 mCi) of the tracer. Participants were scanned for 30 min and six 5 min frames were recorded. Body weight and injected-dose corrected frames were co-registered to one another to reduce motion effects and the five frames were averaged to a single 30 min PET image set for each participant.

#### Magnetic resonance imaging (MRI) acquisition

Structural magnet resonance imaging (MRI) datasets acquired closest to the date of the 18F-AV1451 PET scan were downloaded from the ADNI database for co-registration with the 18F-AV1451 PET scan.

Structural MRIs were acquired on 3T scanners using a high-resolution T1-weighted magnetization prepared rapid gradient echo (MPRAGE) sequence with the following parameters: TR = 2300, TE = minimum full echo, TI = 900, FOV = 208 × 240 × 256 mm, and 1 × 1 × 1 mm resolution.

#### Image preprocessing

Individual 18F-AV1451 PET scans were co-registered to the individual anatomical MPRAGE scan. Each MPRAGE scan was segmented into three tissue classes [i.e., gray matter, white matter, and cerebral spinal fluid (CSF)] using the segmentation function of the partial volume effects correction in brain PET toolbox implemented in SPM 12 (PETPVE12; Gonzalez-Escamilla et al., 2017). Importantly, for each individual MPRAGE scan deformation fields for each individual were derived from Diffeomorphic Anatomical Registration Through Exponentiated Lie Algebra (DARTEL) registration to the reference template (IXI550_MNI152.nii) implemented in PETPVE12. Regions of interests (ROI) were overlaid on co-registered 18F-AV1451 PET scans using the deformation field of the individual native PET space.

#### Region of interests selection

**Figure 1.**
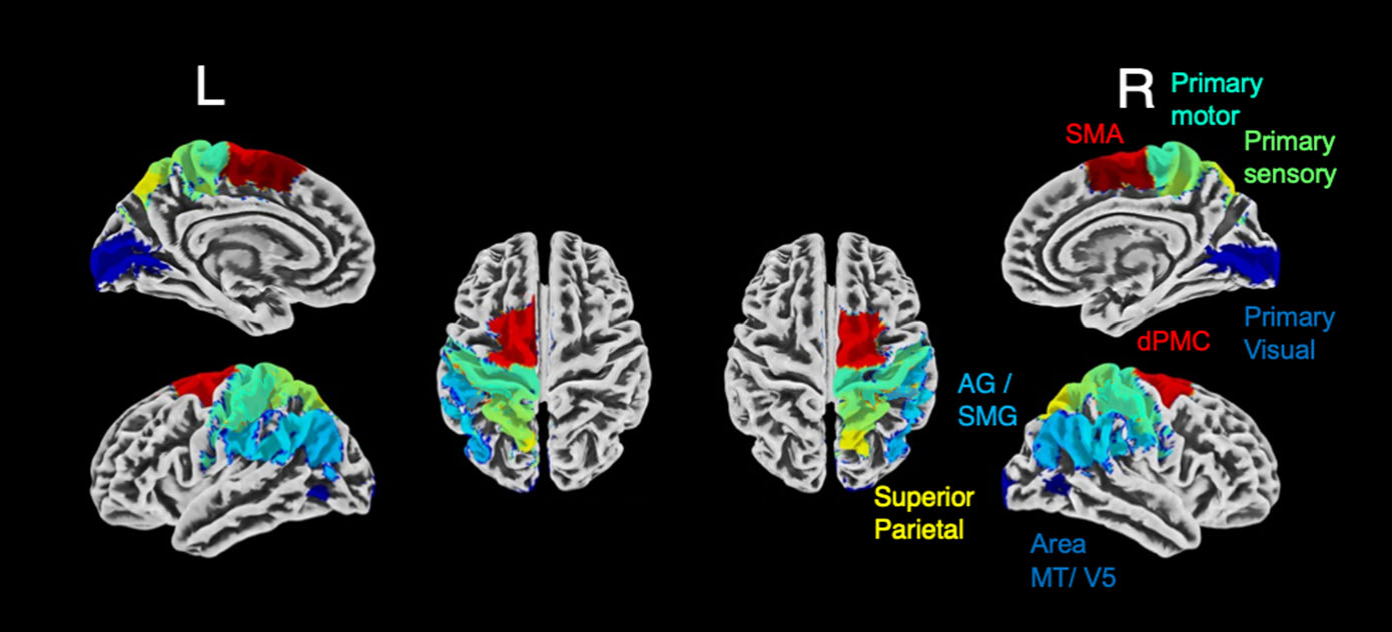
Set of probabilistic regions-of interest (ROIs) used. Cytoarchitectonically regions inferred from the SPM Anatomy toolbox are overlayed on a structural template image in MNI-space using CAT toolbox. L, left hemisphere, R, right hemisphere, ROIs, regions of interest. Colors indicate different label number within the probabilistic motor ROIs, which are exemplary color coded on the right hemisphere.

SPM Anatomy Toolbox Version 3 implemented in SPM 12 (https://www.fz-juelich.de/en/inm/inm-7/resources/jubrain-anatomy-toolbox) was used to create an anatomical mask of the left and right cortical and primary motor regions associated with main motor function and of primary visual, primary sensory as control regions. As there are some known lateralization differences in motor regions (Mutha et al., 2012), we decided to separate the regions by hemisphere. Table 1 lists the derived ROIs and their respective nomenclature in the Julich Brain cytoarchitectonic atlas (Eickhoff et al., 2005) and a display of the examined regions is depicted in Figure 1.

**Table 1.** Cytoarchitectonically mapped regions of the motor network and the corresponding Broadman area in the Julich Brain Atlas

Anatomical networks/regions	Region	Definition Julich Brain areas
Primary regions		
	Primary motor	4a, 4p
	Primary sensory (S1)	1, 2, 3a, 3b
	Primary visual	HOc1
Dorso-dorsal network		
	Dorsal premotor cortex	6d1, 6d2, 6d3
	Superior parietal lobe	5ci, 5l, 5m 7a, 7m, 7p, 7pc
Common network		
	Angular gyrus	PGa, PGp
	Lateral occipital cortex/extra-striate body area	V5
Supramarginal gyrus	SMG	PFcm, PFm, PFop, PFt
Supplementary motor area	SMA	6mc, 6mr

Additionally regions were categorized based on the spatial placement in the motor networks.

#### Standard uptake value ratio quantification

Region-specific standard uptake values (SUV) were extracted from 18F-AV1451 PET scans and from the vermis reference region for each individual subject in native space (see Table 1). To create standardized uptake value ratios (SUVRs), region-specific SUVs were divided by SUVs extracted from the vermis reference region, known to show little specific binding. Of note, the vermis was selected as a reference region due to its lack of involvement in limb motor function, unlike other subregions of the cerebellum (Klein et al., 2016).

#### Statistical analysis

Potential differences in continuous demographic variables (see Table 2) were statistically assessed using univariate analyses of variance (ANOVAs) with group as a factor, whereas categorical variables were examined by chi-square (*X*^2^) test to assess group specific differences in the frequency distribution. The significance thresholds for these descriptive analyses were set at *p* < 0.05.

**Table 2.** Demographic data on included cohort

	HC (*N* = 26)	MCI+ (*N* = 84)	DAD+ (*N* = 25)
Age	73.53 (8.1)	68.61 (5.4)	68.68 (6.3)
Sex (% male)	53	56	68
MMSE	28.7 (1.5)	27.7 (2.0)	22.6 (2.7)
APOE (% carrier)	23%	23%	28%

Data are presented as mean (SD) for continuous variables and percentage for categorical variables. DAD, dementia of the Alzheimer's disease type; APOE4, apolipoprotein E4; HC, healthy controls; MCI, mild cognitive impairment; MMSE, mini mental state examination. “+” Indicates that individuals were amyloid positives.

To evaluate potential differences between groups across the regions known to underlie motor function, we statistically modelled a repeated measurement ANOVA with group as a between-subject factor and age as a covariate. Within subject factors were hemisphere (left and right) and region (seven primary and higher-order motor ROIs and two control regions). Potential interaction effects of the factors group and region were then explored for each region by a one-way ANOVA with the between subject factor group. If a significant main effect of group was detected for a given region by the one-way ANOVA, planned *post hoc* comparisons were executed to further detail the potential source of the group differences in that region. This allowed us to significantly reduce the number of planned comparisons, despite our hypothesis driven ROI approach. Significance threshold was set at *p* < 0.05.

Supportive evidence for the contribution of tau pathology in anatomical motor regions towards disease severity was evaluated by partial correlation analyses, quantifying the relationship of ADAS-Cog-13 with the tau signal in the respective praxis regions, controlling for diagnostic group and age. Confidence-Intervals for correlation coefficients were calculated using a bootstrap procedure (1,000 repetitions) implemented in SPSS v27. All other statistical analyses were performed in SPSS v27 as well.

## Results

A total of *N* = 135 subjects were included in the analysis. Demographic information on these subjects is summarized in Table 2. Age and MMSE were significantly different among the groups (*F*_(Age)__(2,132)_ = 6.515, *p *= 0.002; *F*_(MMSE)__(2,132)_ = 68.276, *p *< 0.001), whereas the APOE epsilon 4-allele carrier status and sex distribution were not (*X*^2^_(APOE)__(2,*N* = 135)_ = 7.5, *X*^2^_(Sex)__(2,*N* = 135)_ = 1.3 *p *> 0.05). Due to the group differences in age, age was entered as a covariate in subsequent analysis.

**Figure 2.**
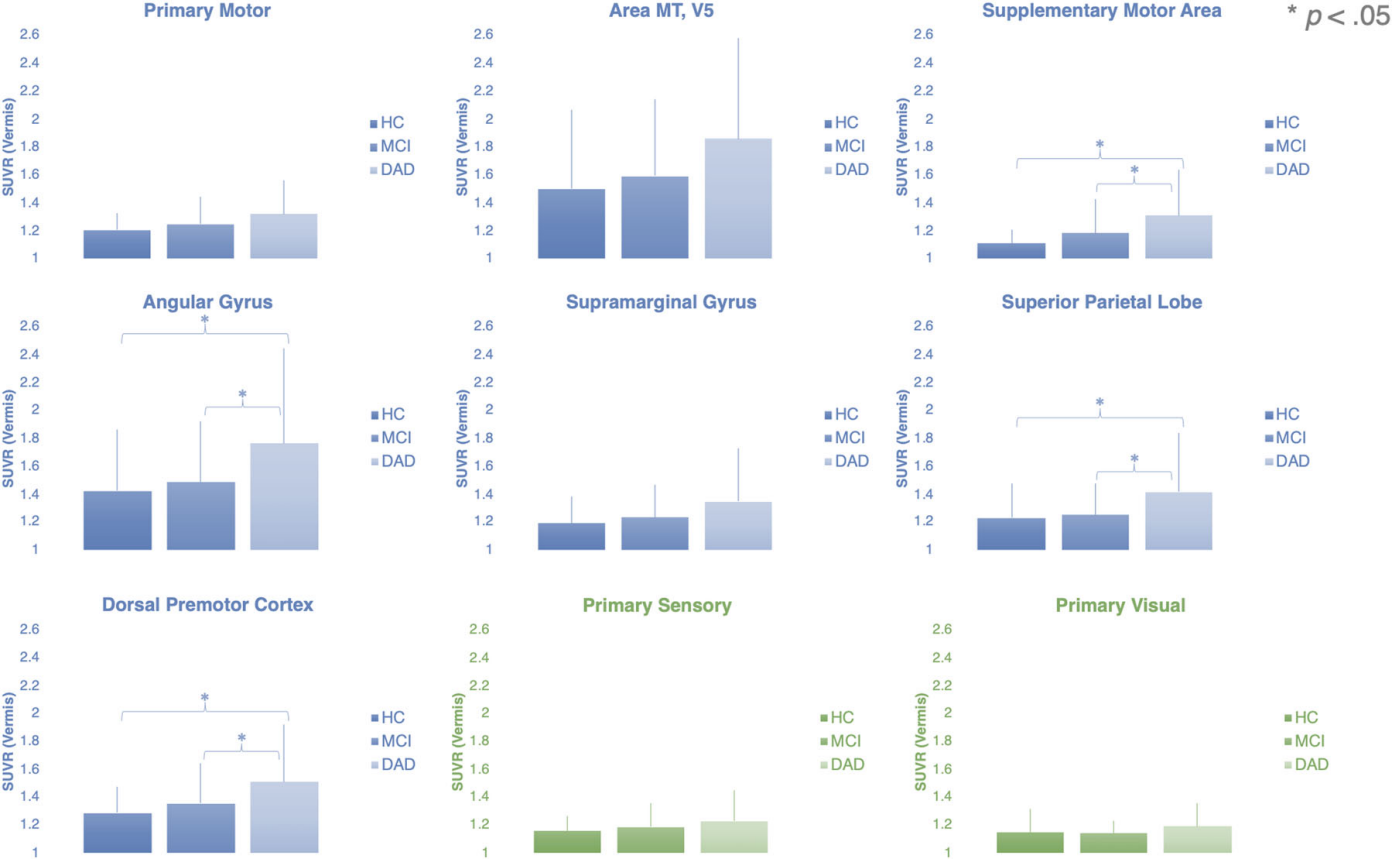
Association of tau pathology in higher-order motor regions with disease category. The mean tau SUVRs for the nine regions are shown collapsed over both hemispheres. The graphs illustrate the significant region × group interaction, since a significant group effect for the mean tau SUVRs was observed in four regions (SMA, supplementary motor area; AG, angular gyrus; SPL, superior parietal lobe; and DPMC, dorsal premotor cortex), while the tau SUVRs did not differ significantly between the three groups in the other five regions (primary motor, area MT/V5, supramarginal gyrus, primary sensory, and primary visual). HC, healthy controls; MCI, mild cognitive impairment; DAD, dementia of the Alzheimer's disease type. * Indicates *p* < 0.05 for the planned *post hoc* comparisons employing *t* tests.

In the repeated measurement ANOVA, we identified a significant Region × Group interaction (*F*_(Region × Group) (2,132)_ = 3.84, *p *< 0.024), while none of the remaining main effects or interactive effects reached significance (for details, see Table 3). Since neither an interaction nor a main effect of hemisphere was detected, we subsequently averaged SUVR values across the left and the right hemisphere in each region. Further interrogation of the region × group interaction using one-way ANOVAs with the between-subject factor group for each of the nine regions, revealed that four of the nine regions (i.e., the supplementary motor area (*p* = 0.011), the angular gyrus (*p* = 0.027), the superior parietal lobe (*p* = 0.027), and the dorsal premotor cortex (*p* = 0.027)) showed a significant main effect of group, while the other five regions did not (see Table 4). We further analyzed the group differences in the four regions using *post hoc* comparisons. Interestingly, in all of these regions the pattern of differences across the groups was similar (see Fig. 2 and Fig. S1). Specifically, the four higher-order motor regions were characterized by higher tau SUVRs in DAD patients compared to both HC and MCI patients. The results are summarized in Table 5.

10.1523/ENEURO.0242-23.2023.f1-1 Figure S1 **Association of tau pathology in higher-order motor regions with disease category in a box-plot diagram. Blue: Motor Regions; Green: Control Regions.** The mean tau SUVRs for the nine regions are shown collapsed over both hemispheres. The graphs illustrate the significant Region × Group interaction, since a significant group effect for the mean tau SUVRs was observed in four regions (SMA; Supplementary Motor Area, AG; Angular Gyrus, SPL; Superior Parietal Lobe, and DPMC; Dorsal Premotor Cortex), while the tau SUVRs did not differ significantly between the three groups in the other five regions (Primary Motor, Area MT/V5, Supramarginal Gyrus, Primary Sensory, and Primary Visual). HC = Healthy Controls, MCI = Mild Cognitive Impairment, DAD = dementia of the Alzheimer's disease type. * indicates p < 0.05 for the planned post-hoc comparisons employing t-tests. Download Figure 1-1, DOCX file.

**Figure 3.**
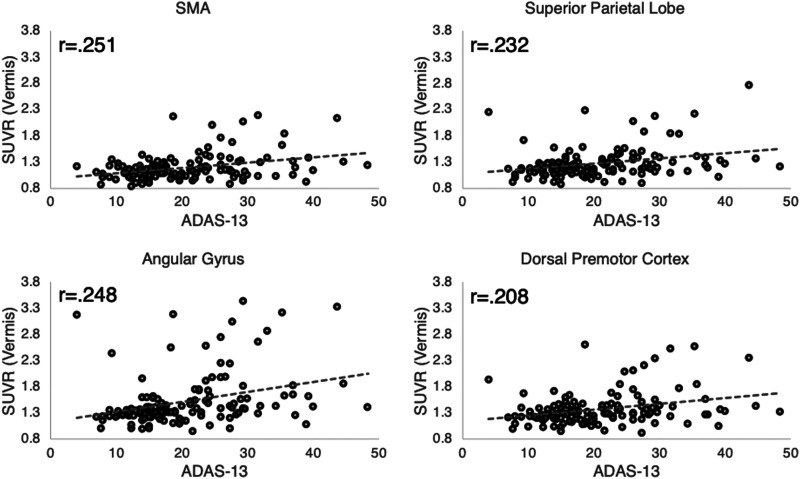
Correlation between tau pathology in higher-order motor regions and cognitive dysfunction. Displayed are correlation coefficients from the partial correlation analysis, examining tau pathology in motor regions and performance on the Alzheimer's Disease Assessment Scale-Cognitive Subscale (ADAS-Cog-13) for all participants (*n* = 135). SMA, supplementary motor area.

**Table 3.** Overall results of the repeated measurement ANOVA, including the factors of group, age, region, and hemisphere and their respective interactions

Repeated measurement ANOVA				
	*df*	*F*	*η_p_* ^2^	*p*
Between-subject effects				
Age	1	0.335	0.003	0.564
Group	**2**	**3.55**	**0.051**	**0.032**
Within-subject effects				
Hemisphere	1	1.34	0.010	0.249
Hemisphere × age	1	1.07	0.008	0.301
Hemisphere × group	2	0.162	0.002	0.850
Region	1	0.272	0.002	0.603
Region × age	1	0.135	0.001	0.714
Region × group	**2**	**3.84**	**0.055**	**0.024**
Hemisphere ×region	1	0.949	0.007	0.332
Hemisphere × region × age	1	0.950	0.007	0.332
Hemisphere × region × group	1	0.127	0.002	0.880

df, degrees of freedom. Bold font indicates significant effects.

**Table 4.** Summary of the one-way ANOVA evaluating the group × region interaction with the significant main effect of group displayed

Between-group differences	*df*	*F*	*η_p_* ^2^	*p*
Primary motor	2	2.11	0.031	0.125
Area MT, V5	2	2.64	0.039	*0.075*
SMA	**2**	**4.68**	**0.066**	**0.011**
Angular gyrus	**2**	**3.69**	**0.053**	**0.027**
SMG	2	2.54	0.037	*0.083*
SPL	**2**	**3.71**	**0.053**	**0.027**
DPMC	**2**	**3.73**	**0.053**	**0.027**
Primary sensory	2	1.17	0.017	0.331
Primary visual	2	1.57	0.023	0.211

The order of regions in this table follows the display order in Figure 2. SMA, supplementary motor area; SMG, supramarginal gyrus; SPL, superior parietal lobe; DPMC, dorsal premotor cortex; df, degrees of freedom. Regions in bold font show significant effects.

**Table 5.** Results of the planned *post hoc* comparison in regions with a significant group effect (see Table 4)

Region	DAD versus	HC/MCI	Mean difference (SE)	Significance	Confidence level	
					Lower bound	Upper bound
SMA						
SMA	DAD	HC	0.204 (0.067)	**0.031**	0.069	0.338
SMA	DAD	MCI	0.128 (0.055)	**0.021**	0.019	0.237
Angular gyrus						
AG	DAD	HC	0.340 (0.139)	**0.015**	0.064	0.617
AG	DAD	MCI	0.280 (0.113)	**0.016**	0.055	0.505
SPL						
SPL	DAD	HC	0.185 (0.078)	**0.021**	0.028	0.340
SPL	DAD	MCI	0.164 (0.064)	**0.012**	0.037	0.290
DPMC						
DPMC	DAD	HC	0.224 (0.085)	**0.010**	0.055	0.393
DPMC	DAD	MCI	0.155 (0.069)	**0.027**	0.017	0.290

HC, healthy controls; MCI, mild cognitive impairment; DAD, dementia of the Alzheimer's disease type; SE, standard error; SMA, supplementary motor area; SMG, supramarginal gyrus; SPL, superior parietal lobe; DPMC, dorsal premotor cortex. Bold numbers denote significant effects (*p* < .05).

**Table 6.** Partial correlation on relationship between ADAS-Cog-13 and motor regions showing a significant main effect of group on tau SUVR

Partial correlation coefficients (age, group)			SMA	Angular gyrus	SPL	DPMC
ADAS-Cog-13	Correlation		**0.251**	**0.248**	**0.232**	**0.208**
	Significance		**0.004**	**0.004**	**0.007**	**0.017**
	df		130	130	130	130
	95% confidence interval	Lower	0.067	0.070	0.035	0.044
		Upper	0.410	0.427	0.418	0.397

SMA, supplementary motor area; SPL, superior parietal lobe; DPMC, dorsal premotor cortex; df, degrees of freedom, 95% confidence interval based on bootstrapping (1,000 repetitions) implemented in SPSS v27. Bold numbers denote significant effects (*p* < .05)

Although area MT/V5 (*p* < 0.075) and the supramarginal gyrus (*p* < 0.083) showed trend-significant group effects only, a similar pattern was observed in these two regions. Albeit not significant, DAD patients had higher tau SUVRs in area MT/V5 and the supramarginal gyrus than MCI patients and HC (see Fig. 2, and Fig. S1). Importantly, none of the primary regions for motor, sensory, or visual processing showed a significant group effect. Moreover, we observed relatively low tracer retention in these three regions (see Fig. 2). These results confirm the regional specificity of tau pathology in higher-order motor regions, whereas primary regions in DAD and MCI patients show negligent levels of tau burden, which are indistinguishable from HC.

To evaluate the potential impact of in vivo tau pathology in (higher-order) motor regions on a general cognitive dysfunction, we examined the partial correlation between ADAS-Cog-13 and tau SUVR in those motor regions that displayed a significant group effect (i.e., SMA, angular gyrus, superior parietal lobe, and dorsal premotor cortex). Partial correlations were corrected by age and group. Indeed, we found moderate, but significant, positive correlation coefficients of tau SUVR and increases in ADAS-Cog-13 across the cohort (see Table 6 and Fig.3). Specifically, SMA showed the strongest correlation (*r* = 0.251), followed by the angular gyrus (*r* = 0.248) and superior parietal lobe and dorsal premotor cortex with the smallest correlation coefficients (*r* = 0.232 and *r* = 0.208)

## Discussion

The goal of the study was to examine if relevant in vivo tau pathology in prodromal and clinical stages of AD is present in regions of the motor network. We chose cytoarchitectonically parcellated regions of the Julich Brain Atlas to allow for distinct and accurate anatomical mapping of motor regions. The Julich Brain Atlas has advantages due to its precision and integration of individual variations of stereotaxic space (Amunts et al., 2020) including the core regions of the motor network. We found that increased tau pathology in clinical stages of AD was most pronounced in the supplementary motor area, angular gyrus, superior parietal regions, and dorsal premotor cortex, whereas trend-significant effects were observed for movement-related area MT/V5 and supramarginal gyrus. Importantly, all primary regions, including primary motor, primary sensory and primary visual regions did not show significant alterations as a function of clinical diagnosis of AD. For the higher motor regions showing a significant group effect, we identified a significant association of general cognitive dysfunction and increases in tau pathology, irrespective of diagnostic group.

Histopathological studies have suggested that early stages of neurofibrillary tangle protein inclusions are present in the transenthorinal regions (Braak I–II) prior to the involvement of the limbic regions (Braak III–IV) and finally the isocortical regions (V–VI), suggesting an area-specific distribution pattern rather than a random occurrence of protein pathology (Braak and Braak, 1995). With regard to the higher-order motor regions examined here, the majority of regions (i.e., supramarginal gyrus, superior parietal lobe, angular gyrus, premotor areas) have been described to constitute Braak V, whereas regions such as area MT/V5 and primary sensory and motor neocortex are thought to be part of Braak VI. This is in correspondence to the presented findings here, as elevated tau pathology in higher-order motor regions (i.e., regions included in Braak V) were most visible in the clinical disease stages of DAD followed by prodromal stages of AD compared to healthy controls. Additionally, no significant group differences in primary regions were observed indicating the lack of relevant tau pathology in these regions which confirms the stereotypical pattern of tau in histopathological data.

It is important to note that the driving concept of the histopathological approaches taken by neuropathologist was to describe the anatomical extent of the disease, not primarily the intensity or concentration of the protein inclusions observed (Del Tredici and Braak, 2020). In vivo PET imaging of protein pathology can now broaden the scope of our disease understanding by including both extent and intensity and temporal course/symptom correlation to inform on the disease processes. The observed increased uptake pattern in late-stage disease regions can alternatively be explained by different maturity states of tau protein pathology that are detected with 18F- AV1451. Specifically, it is possible that pre-tangle pathology is evolving in higher-order motor regions, as it has been shown that 18F- AV1451 has a higher affinity to pre-tangle formation of tau, which may be the source of signal we observed here (Braak et al., 2011; Wren et al., 2018).

From a motor systems perspective, our data provide in vivo evidence for a particular involvement of higher motor regions in the spreading of tau pathology with symptomatic disease progression, which is a novel finding. Lesion studies, mainly performed in stroke patients, indicate that cognitive-motor processes are organized in dorso-dorsal, ventro-dorsal, and ventral processing streams (Martin et al., 2017). Our results suggest that particular the dorso-dorsal stream (e.g., dorsal premotor cortex, superior parietal cortex) and part of the ventro-dorsal processing stream (e.g., angular gyrus) are affected by tau pathology in AD. Indeed, major higher-order regions of the motor system spanning from motor planning (angular gyrus) to initiation of internally (e.g., SMA) or externally (e.g., premotor cortex) triggered movements show a significant correlation of increased tau pathology with increasing clinical disease severity. These findings indicate that the motor system is not spared from aggregation of tau pathology even in relatively early stages of disease. It is important to note, that some of the investigated regions have been shown to subserve additional function, such as working memory processing or distributing attentional demands or semantic processing such as the superior parietal lobule or the angular gyrus (Horwitz et al., 1998; Alahmadi, 2021). Given that such functions become gradually impaired with clinical disease severity, it is feasible that tau pathology in these particular regions may impair cognitive processes beyond motor function alone. The distinctiveness and importance of the shown vulnerability of motor regions to the pathophysiological build-up of tau pathology is, however, well represented in the supplementary motor area, a region strictly responsible for motor function (Tanji, 1994; Tanji and Shima, 1996; Côté et al., 2020; Rahimpour et al., 2022).

The consequences of these motor-associated tau aggregates with regard to potential impairment of higher-order motor-cognitive functions are yet unknown, as specific praxis functions were not examined in this cohort. However, accumulating evidence suggests that specific praxis functions in AD are impaired (Johnen et al., 2015; Yliranta et al., 2023b). Interestingly, some studies suggest that limb apraxia and verbal memory may indeed improve differential diagnosis of DAD compared to other psychiatric etiologies of dementia (Yliranta et al., 2023a). The current study suggests that the potential effects of tau deposition in motor areas on specific motor functions should be further examined. For this reason, our group is currently investigating in a prospective trial, if the clinical symptomatology of praxis dysfunction in DAD is associated with elevated tau pathology in regions subserving higher-order motor function. This may add evidence to the established observation of a close link between the dominant clinical symptoms and the peak regions of the most elevated tau pathology in both typical and atypical AD (Dronse et al., 2016; Ossenkoppele et al., 2016; Bejanin et al., 2017; Bischof et al., 2017). Such imaging-based approaches may also allow to examine in vivo why praxis dysfunction appears to be a later symptom in the sporadic disease cascade only, whereas in early-onset disease cases it has been suggested to be an early behavioral symptom (Yliranta et al., 2023b).

A recent observation which has elucidated our understanding of AD heterogeneity is the presence of endophenotypes based on tau PET scans of 18F-AV1451 (Vogel et al., 2021). Briefly, the authors observed several subgroups of tau deposition patterns: a tau pattern most accurately described by elevated limbic tangle deposition (S1, 33% of the AD sample), a predominant posterior occipital and gradually anterior expanding type (S3, 30%), a hemispheric dominant temporo-parietal phenotype (S4, 19%) and, finally, a medial temporal sparing phenotype, with dominantly parietal lobe involvement (S2, 18%). Importantly, the investigated phenotypes were characterized by different disease onsets (e.g., S1 and S3 late disease onset), and clinical presentation (e.g., S3, visuospatial impairment; S2, dysexecutive impairment; S4, multi-domain impairment) and disease progression (e.g., S3, slow progressing; S4, fast progressing). Overall, these data suggest that differential spatial patterns of tau pathology can influence disease trajectories. It will be interesting to further investigate if clinical profiles of praxis dysfunction align with differential patterns of tau deposition in higher-order motor regions and if apraxia could be an additional feature that complements the clinical characteristics of the described tau PET endophenotypes.

Finally, we observed that increased tau deposition in higher-order motor regions was associated with general cognitive dysfunction. Although the correlation coefficients were moderate in size, they provide support for the idea that tau pathology in higher-order motor regions contributes to the cognitive decline in patients with Alzheimer's pathology, over and above the clinically defined diagnostic group.

Some limitations of our current approach should be acknowledged. First of all, although we detected elevated tau pathology in higher-order motor regions, the clinical relevance to the commonly observed praxis deficits in AD could not be interrogated due to the lack of detailed characterization of praxis function in this cohort. Additionally, only cross-sectional inferences on the potential group differences of tau pathology in motor regions can be drawn from this study. It would be interesting to examine how tau pathology in motor regions advances in individuals who progress along on the continuum of AD and how this is associated with accompanying deterioration of cognitive function in the prodromal and the advanced stages of DAD.

A drawback of the tau tracer 18F-AV1451 is the limited ability to accurately measure tau pathology in basal ganglia due to the concomitant nontau-specific binding, presumably to MAO-A and MAO-B in this brain region (Bischof et al., 2017; Leuzy et al., 2019; Bischof, 2022). The basal ganglia are, however, instrumental for motor control and other next-generation tracers void of nontau-specific target binding in basal ganglia, may be better suited for further assessment of tau-specific basal ganglia uptake in a motor-centered regional analysis.

In sum, we investigated patterns of elevated tau pathology in motor regions in a large sample of patients with MCI and DAD and observed significant increases of tau pathology in higher-order motor processing regions as compared to HC. Importantly, the level of tau pathology in the investigated pathways was associated with decreases in cognitive function. This indicates a clinicopathological link between tau in motor-cognitive areas and advances our knowledge beyond the known relationship of tau patterns in medial temporal regions and memory in AD.

## Synthesis

Reviewing Editor: Laura Volpicelli-Daley, The University of Alabama at Birmingham Decisions are customarily a result of the Reviewing Editor and the peer reviewers coming together and discussing their recommendations until a consensus is reached. When revisions are invited, a fact-based synthesis statement explaining their decision and outlining what is needed to prepare a revision will be listed below. The following reviewer(s) agreed to reveal their identity: NONE. Note: If this manuscript was transferred from JNeurosci and a decision was made to accept the manuscript without peer review, a brief statement to this effect will instead be what is listed below.

Intracellular accumulation of tau proteins is a known feature of Alzheimer's Disease (AD), but their progression in motor cortical regions is not well described. In this manuscript, the authors analyze available tau PET scan data in order to quantify the relationship between tau prevalence in motor regions with symptoms of AD in three different diagnostic groups of subjects. Using regression analyses, they conclude that tau accumulation in motor cortex correlates with severity of AD-related cognitive impairment. While the results are of potential significance, a paucity of details makes their interpretation somewhat difficult.

Major points:

1. More details needed about the method used for conducting the partial correlation and multiple regression analyses, especially since they constitute the bulk of the results.

a. In line 197, the authors mention general linear models - were any non-linear link functions applied between the regressors and the dependent variable?

b. I am not sure what the authors mean by "univariate simple regressions with group as a factor" (line 194). Since variables like group and hemisphere are categorical, linear regression seems to be an unconventional choice here. Do the authors mean that linear fits were made on the data shown in Figure 2? More clarification is needed.

c. It is unclear which statistical test was used to calculate the p-values shown in Figure 2. Ideally, the details of the test(s) should be mentioned in the figure legend itself. Moreover, it is unclear if the authors employed a test appropriate for making multiple comparisons across all 3 groups.

2. In general, the way the results are presented could be much improved.

a. For instance, it would be particularly helpful to show more scatterplots and linear fits, at least for all of the univariate regression analyses.

b. It would also be useful to provide some visualization of the results of the multiple regression analysis provided in table 3 to make it easier to parse the numbers.

c. The results section is mostly written as a list of numerical results without any contextualization as to their potential significance.

Minor points:

1. In Figure 1, the different colored regions should be labeled.

2. It is unclear from the text if the patients in the MCI group showed any progressive deterioration in cognitive function.

3. There are a number of typographical and other errors, for instance, lines 109-111.

4. Several abbreviations are not introduced at first use.

5. It would be useful to specify in the methods the acceptable time window between an MRI scan and an 18F-AV1451 scan.
