# Supplementary material for: Affection of Motor Network Regions by Tau Pathology Across the Alzheimer's Disease Spectrum
Source: eNeuro. 2024 Jan 16;11(1):ENEURO.0242-23.2023. doi: 10.1523/ENEURO.0242-23.2023 (PMC10849022; doi:10.1523/ENEURO.0242-23.2023)
Supplement: Figure S1 — Association of tau pathology in higher-order motor regions with disease category in a box-plot diagram. Blue: Motor Regions; Green: Control Regions. The mean tau SUVRs for the nine regions are shown collapsed over both hemispheres. The graphs illustrate the significant Region × Group interaction, since a significant group effect for the mean tau SUVRs was observed in four regions (SMA; Supplementary Motor Area, AG; Angular Gyrus, SPL; Superior Parietal Lobe, and DPMC; Dorsal Premotor Cortex), while the tau SUVRs did not differ significantly between the three groups in the other five regions (Primary Motor, Area MT/V5, Supramarginal Gyrus, Primary Sensory, and Primary Visual). HC = Healthy Controls, MCI = Mild Cognitive Impairment, DAD = dementia of the Alzheimer's disease type. * indicates p < 0.05 for the planned post-hoc comparisons employing t-tests. Download Figure 1-1, DOCX file. [file eneuro-11-ENEURO.0242-23.2023-s001.docx]

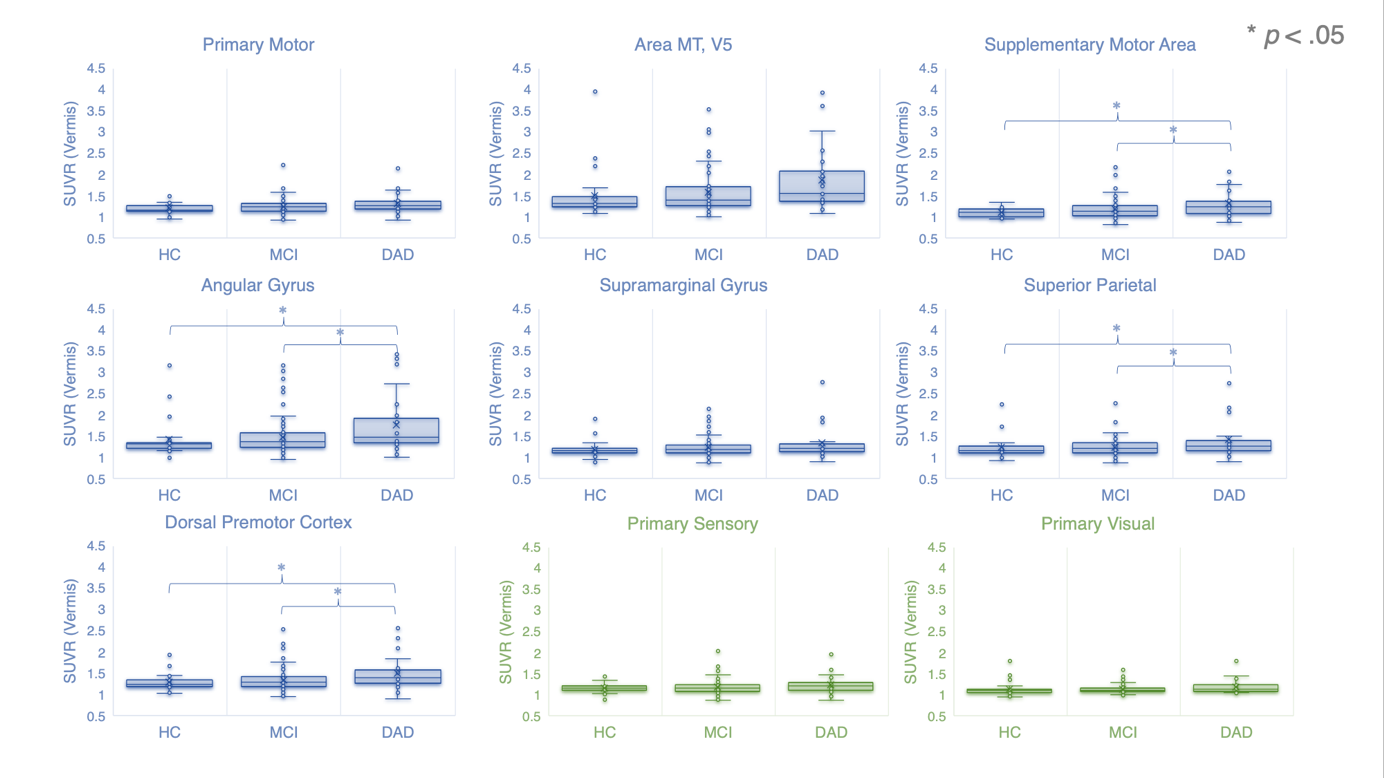
Figure S1. ***Association of tau pathology in higher-order motor regions with disease category in a box-plot diagram. Blue: Motor Regions; Green: Control Regions.*** *The mean tau SUVRs for the nine regions are shown collapsed over both hemispheres. The graphs illustrate the significant Region × Group interaction, since a significant group effect for the mean tau SUVRs was observed in four regions (SMA; Supplementary Motor Area, AG; Angular Gyrus, SPL; Superior Parietal Lobe, and DPMC; Dorsal Premotor Cortex), while the tau SUVRs did not differ significantly between the three groups in the other five regions (Primary Motor, Area MT/V5, Supramarginal Gyrus, Primary Sensory, and Primary Visual). HC = Healthy Controls, MCI = Mild Cognitive Impairment, DAD = dementia of the Alzheimer’s disease type. * indicates p<0.05 for the planned post-hoc comparisons employing t-tests.*
